# Interfacial Asymmetry and Surface Roughness Enhanced Topological‐Hall‐Like Transport in SRO/BTO/SRO Trilayers

**DOI:** 10.1002/advs.78126

**Published:** 2026-10-06

**Authors:** Sagar E. Shirsath, Yunlong Sun, Xichao Zhang, Ji Zhang, Akash V. Fulari, Zizheng Song, Yee Yan Tay, Yin Yao, Zibin Chen, Sean Li, Xiaoxi Liu, Danyang Wang

**Affiliations:** ^1^ School of Materials Science and Engineering The University of New South Wales Sydney New South Wales Australia; ^2^ Department of Electronic and Computer Engineering The Hong Kong University of Science and Technology Hong Kong China; ^3^ Symbiosis Centre for Nanoscience and Nanotechnology Symbiosis International (Deemed University) Pune Maharashtra India; ^4^ State Key Laboratory of Ultra‐Precision Machining Technology Department of Industrial and Systems Engineering The Hong Kong Polytechnic University Hong Kong China; ^5^ Research Institute for Advanced Manufacturing Department of Industrial and Systems Engineering The Hong Kong Polytechnic University Hong Kong China; ^6^ Facility for Analysis, Characterisations, Testing and Simulations (FACTS) Nanyang Technological University Singapore Singapore; ^7^ Electron Microscope Unit Mark Wainwright Analytical Centre UNSW Sydney The University of New South Wales Sydney New South Wales Australia; ^8^ Department of Electrical and Computer Engineering Shinshu University Nagano Japan

**Keywords:** micromagnetic simulation, oxide trilayer engineering, SrRuO_3_–BaTiO_3_ heterostructure, surface roughness, Topological‐Hall‐like effect

## Abstract

Engineering heterostructure architecture in complex oxides offers a promising route for tuning emergent topological transport. Here, we show that replacing a single‐interface BaTiO_3_/SrRuO_3_ (BTO/SRO) bilayer with an SRO/BTO/SRO trilayer produces a pronounced enhancement of the topological‐Hall‐like response. The trilayer exhibits a maximum extracted topological‐Hall‐like resistivity of 0.8 µΩ cm, approximately 2.5 times larger than that of the bilayer, together with a broad stability window from 5 to 90 K. Atomic‐scale strain and polarization/displacement mapping reveal structurally and electrostatically nonequivalent upper and lower SRO environments, while AFM shows substantially greater morphological modulation in the trilayer. Micromagnetic simulations demonstrate that noncoplanar magnetic configurations are physically permissible over a finite parameter range in this architecture. However, because the two nonequivalent SRO layers may also contribute distinct anomalous Hall channels, the microscopic origin of the hump‐like Hall contribution cannot be uniquely assigned from the present transport measurements. The experimentally established result is that the trilayer architecture substantially enhances and broadens the topological‐Hall‐like response compared with the bilayer.

## Graphical abstract

Trilayer oxide architecture introduces interfacial asymmetry and surface roughness in SRO/BTO/SRO heterostructures, creating a locally modulated polar and magnetic landscape. This design enhances the topological‐Hall‐like response, achieving a broad 5–90 K stability window and a maximum ρ_THE_ of 0.8 µΩ cm, demonstrating an effective route to amplify chiral transport.

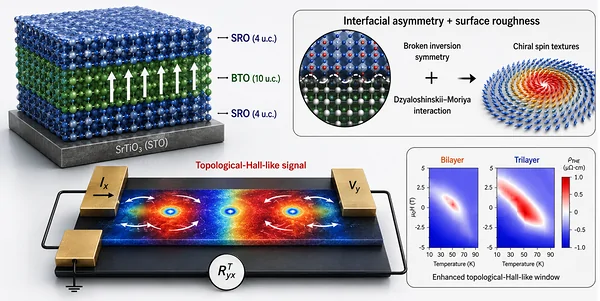

## 1 Introduction

Topological spin textures, such as magnetic skyrmions, have attracted considerable attention because they could generate real‐space Berry curvature, give rise to unconventional transport signatures, and offer a promising basis for low‐power and nonvolatile spintronic devices [1, 2, 3, 4, 5, 6]. Among these signatures, the topological‐Hall‐like response (THE) is widely used as an electrical probe of noncoplanar magnetic textures [1, 2, 3, 7]. In thin films and heterostructures, the stabilization of such chiral spin states generally requires a broken inversion symmetry together with a sizable spin–orbit coupling, which produces a finite Dzyaloshinskii–Moriya interaction (DMI) [2, 3, 7, 8, 9]. Complex oxides are particularly attractive in this regard because lattice, charge, orbital, and polar degrees of freedom can all be tuned at the unit‐cell scale through heterointerface engineering [8, 10, 11, 12, 13, 14].

SrRuO_3_ (SRO) is a compelling platform for this purpose because it combines itinerant ferromagnetism with strong spin–orbit coupling and pronounced sensitivity to epitaxial distortion [7, 15, 16, 17, 18, 19]. Interface engineering has already been shown to induce THE in ultrathin SRO, as demonstrated in SRO/SrIrO3 bilayers and electrically tunable other oxide heterostructures [7, 20, 21, 22, 23, 24, 25]. More recently, topological‐Hall‐like signatures have also been observed in single‐layer or weakly capped SRO films, where interfacial octahedral tilting, structural rumpling, and local inversion‐symmetry breaking provide the microscopic origin of DMI [15, 17, 26]. These studies establish that THE in SRO is governed not simply by the nominal layer composition, but by the local structural asymmetry of the magnetic layer and its interfaces [18, 23, 25].

An important advance in this direction was the demonstration that ferroelectric BaTiO_3_ (BTO) can induce and tune chiral magnetism in ultrathin SRO through a ferroelectric proximity effect [10, 19]. In BTO/SRO bilayers, ferroelectrically driven ionic displacements penetrate into interfacial SRO, generate a finite Ru─O rumpling, and produce a sizable interface‐induced DMI capable of stabilizing skyrmion‐like topological spin textures and an accompanying THE [10, 25]. At the same time, studies on SRO/BTO/SRO heterostructures have shown that the ferroelectric response of BTO is highly sensitive to interface termination and local structural symmetry [27]. Mixed or asymmetric terminations can produce pinned dipoles and spatially inhomogeneous polarization, whereas atomically controlled interfaces preserve robust ferroelectricity at ultrathin thickness [27]. These results indicate that the symmetry of an SRO/BTO/SRO trilayer should not be judged only from its nominal stacking sequence. Although an ideal trilayer with identical upper and lower interfaces would be relatively symmetric, the experimentally realized structure can become vertically asymmetric because the two SRO layers experience different boundary conditions. In the present case, the lower SRO layer is clamped by the STO substrate, whereas the upper SRO layer is grown on BTO and terminates at the free surface. Such asymmetric boundary conditions can modify local strain, polar displacement, octahedral distortion, and hence the interfacial magnetic energy landscape.

It is important to recognize that a hump‐like Hall response does not uniquely establish a real‐space topological spin texture. In ultrathin SRO systems, similar Hall features may arise from the superposition of anomalous Hall contributions associated with magnetically or electronically nonequivalent conducting regions [28, 29]. In particular, parallel SRO channels with different anomalous Hall coefficients and coercive fields can generate hump‐like transverse‐voltage loops even without skyrmion formation [28, 30]. Conversely, direct real‐space and atomic‐scale studies of ferroelectric/SRO and related SRO heterostructures have demonstrated that interfacial polar displacement and broken inversion symmetry can produce DMI‐related noncoplanar magnetic configurations [31, 32]. Therefore, the present work focuses on the experimentally established architecture‐dependent enhancement of the Hall anomaly and evaluates whether the accompanying structural and simulated magnetic landscape is physically consistent with a noncoplanar‐spin contribution, without assuming that Hall transport alone uniquely identifies its microscopic origin.

This provides the motivation for examining whether architecture‐induced asymmetry can enhance topological‐Hall‐like transport beyond the single‐interface BTO/SRO limit. Scientifically, the trilayer geometry allows us to test whether dual SRO/BTO‐related interfaces and nonequivalent SRO environments can provide an additional route to tune local inversion‐symmetry breaking and DMI‐related chiral spin transport [33]. Technologically, enhancing the magnitude and temperature/field stability of the topological‐Hall‐like signal is important for improving the electrical detectability and robustness of chiral magnetic states in oxide spintronic heterostructures. The relevant parameters are also experimentally tunable: strain through substrate choice, lattice mismatch, and thickness; polarization through ferroelectric thickness, interface termination, and poling; and morphology through growth temperature, oxygen pressure, and deposition conditions. Therefore, the bilayer–trilayer comparison provides a practical platform for evaluating how vertical architecture can modify the local symmetry environment and enhance topological‐Hall‐like transport.

Here, we compare a BTO(10 u.c.)/SRO(4 u.c.) bilayer with an SRO(4 u.c.)/BTO(10 u.c.)/SRO(4 u.c.) trilayer grown on SrTiO_3_ to determine how heterostructure architecture influences topological transport in ultrathin oxide systems. We find that the trilayer exhibits a markedly enhanced topological‐Hall‐like response, with a maximum THE of approximately 0.8 µΩ cm, approximately 2.5 times larger than that of the bilayer, over a broad temperature window of 5–90 K. By combining Hall transport, TEM‐based strain and polarization/displacement mapping, magnetometry, micromagnetic simulations, and AFM, we demonstrate that the trilayer contains two nonequivalent SRO environments: a substrate‐constrained lower SRO layer and a comparatively relaxed upper SRO layer with greater structural and electrostatic heterogeneity. These observations establish the trilayer as an effective architecture for producing a stronger and broader Hall anomaly than the corresponding bilayer. The observed vertical asymmetry and morphological modulation are correlated with this enhancement, although the microscopic contribution of each structural factor cannot be independently separated in the present study.

## 2 Results and Discussion

### 2.1 Heterostructure Design and Interfacial Symmetry Concept

To investigate how ferroelectric interfacing and magnetic layer configuration affect chiral spin transport in oxide heterostructures, we designed two epitaxial model systems on SrTiO_3_ (STO): a bilayer BaTiO_3_ (BTO, 10 u.c.)/SrRuO_3_ (SRO, 4 u.c.)/STO and a trilayer SRO (4 u.c.)/BTO (10 u.c.)/SRO (4 u.c.)/STO, as illustrated schematically in Figure 1a,b. The purpose of this architecture is to compare a single ferroelectric/ferromagnetic interface with a sandwiched magnetic/ferroelectric/magnetic geometry. In this geometry, the upper SRO layer introduces an additional SRO/BTO interface and may allow indirect interlayer influence through magnetostatic/dipolar fields, strain transfer, and polarization‐mediated boundary conditions across the BTO spacer [34]. Such a comparison is particularly relevant because topological Hall signals in ultrathin SRO‐based oxides are known to be highly sensitive to inversion‐symmetry breaking, interface quality, and octahedral distortions, all of which can be engineered by heterointerface design [10, 35].

**FIGURE 1:**
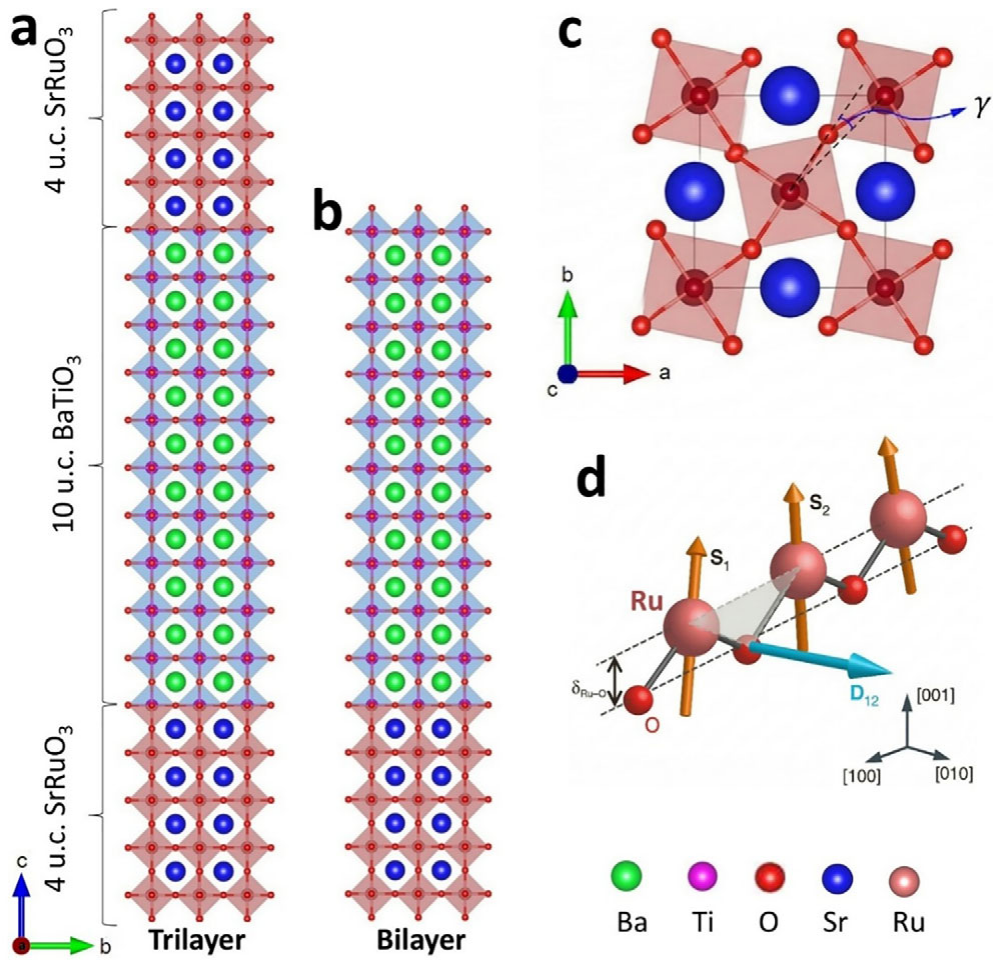
Structural design and interfacial symmetry concept of the bilayer and trilayer heterostructures. (a) Schematic of the bilayer BTO(10 u.c.)/SRO(4 u.c.)/STO. (b) Schematic of the trilayer SRO(4 u.c.)/BTO(10 u.c.)/SRO(4 u.c.)/STO. (c) Schematic of the SRO atomic structure in the ab plane, highlighting the RuO_6_ octahedral rotation. (d) Illustration of the interfacial Dzyaloshinskii–Moriya interaction (DMI) associated with a nonzero Ru–O displacement, where the DMI vector D_12_ is perpendicular to the triangle formed by two neighboring Ru cations and the bridging oxygen ligand.

The physical basis of Figure 1c,d is the emergence of interfacial Dzyaloshinskii–Moriya interaction (DMI) under broken inversion symmetry and strong spin–orbit coupling. In ultrathin SRO, polar distortion and Ru─O rumpling near the SRO/BTO interface can generate finite interfacial DMI and favor noncoplanar magnetic textures [10]. The corresponding magnetic energy may be expressed as [36, 37, 38].

(1) $$\begin{array}{ccc} H & = & -\sum_{\langle i,j\rangle }J_{ij}\;S_{i}\cdot S_{j}+\sum_{\langle i,j\rangle }D_{ij}\cdot \left(S_{i}\times S_{j}\right)-K\sum_{i}{\left(S_{i}^{z}\right)}^{2}\\ & & -\;\mu_{0}\sum_{i}H\cdot S_{i}-\frac{\mu_{0}}{2}\sum_{i}H_{D}\cdot S_{i}\; \end{array}$$

where J_ij_ is the symmetric exchange interaction, **D**_ij_ is the DMI vector, K is the magnetic anisotropy constant, **H** is the applied magnetic field, and ***H***_D_ is the demagnetizing field resulting from the dipolar interaction. The second term is the key ingredient for stabilizing the chiral spin textures with nontrivial topology [38, 39]. In the interfacial geometry relevant here, the DMI vector is set by the broken local symmetry of the Ru‐O‐Ru bond network and is perpendicular to the triangle formed by two neighboring Ru cations and the bridging oxygen ligand, as illustrated in Figure 1d [10, 35]. For a nonzero cation‐oxygen rumpling, the DMI strength scales qualitatively with the interfacial displacement [1], so that larger polar distortion is expected to enhance the tendency toward noncollinear spin configurations [10, 35].

(2) $$|D_{12}|\propto \delta_{\mathrm{Ru}-O}$$

The atomic picture in Figure 1c is also important because the octahedral geometry in ultrathin SRO is not a passive structural detail. Prior studies on SRO thin films and oxide interfaces have shown that octahedral rotation, octahedral tilting, and Ru─O rumpling can directly modify inversion symmetry breaking and therefore the magnitude of the effective DMI [7, 10, 16]. In particular, interfacial oxygen octahedral tilting in ultrathin SRO has been shown to break inversion symmetry and contribute to the formation of topological‐Hall‐like signatures, whereas COBRA and STEM analyses on 4‐u.c. SRO have revealed that the DMI vector is intimately linked to cation‐oxygen rumpling [16, 35]. Thus, the octahedral‐rotation sketch in Figure 1c is not merely illustrative; it represents a structural degree of freedom that can govern the magnetic energy landscape.

The trilayer geometry is expected to differ from the bilayer geometry in at least three ways. First, it contains two SRO/BTO‐related interfaces rather than one, thereby increasing the number of structurally active regions in which the inversion symmetry may be locally broken. Second, previous studies on SRO/BTO/SRO capacitors have demonstrated that the atomic termination sequence in this stack is highly consequential for ferroelectric behavior: symmetric SrO‐TiO_2_ interfaces favor robust and switchable polarization, whereas asymmetric or mixed terminations can produce pinned dipoles and local ferroelectric inhomogeneity [27]. Because interfacial polarization and ionic displacement are closely connected to DMI formation in BTO/SRO heterostructures, the termination‐controlled interfacial structure of SRO/BTO/SRO is directly relevant to the present trilayer design [10, 27]. Third, unlike the bilayer, the trilayer contains two ultrathin ferromagnetic SRO layers separated by a 10 u.c. insulating ferroelectric BTO spacer. Therefore, conventional direct exchange coupling between the two SRO layers is unlikely. Any interlayer influence in the present structure should instead be regarded as indirect and effective, arising mainly from magnetostatic/dipolar interaction, strain transfer, and polarization‐mediated electrostatic boundary conditions across the BTO layer. Thus, Figure 1 presents the physical framework used to examine how dual interfaces, vertical structural asymmetry, polar distortion, and indirect interlayer influence may modify the transverse Hall response. These factors can alter interfacial DMI and the stability of noncoplanar magnetic configurations, but may also generate different magnetic‐switching and anomalous Hall responses in the two SRO layers. The trilayer geometry must therefore be considered as a system in which chiral‐spin‐related and multichannel anomalous Hall contributions may coexist [12, 13, 33, 40].

### 2.2 Enhanced Topological‐Hall‐Like Response in SRO/BTO/SRO Trilayers

The central experimental result of this work is presented in Figure 2, which compares the transverse Hall response of the bilayer BTO(10 u.c.)/SRO(4 u.c.)/STO and the trilayer SRO(4 u.c.)/BTO(10 u.c.)/SRO(4 u.c.)/STO over a broad temperature range. As summarized in the figure set, the bilayer Hall data were measured from 5 to 90 K, whereas the trilayer was tracked from 5 to 100 K. The extracted topological Hall component is further visualized through contour maps in the T‐0H plane and by the temperature dependence of THE. The key observation is that the trilayer exhibits a markedly stronger topological‐Hall‐like response than the bilayer, with a maximum of about 0.8 µΩ·cm, which is approximately 2.5 times larger than that of the bilayer, together with a broader stability window extending from 5 to 90 K.

**FIGURE 2:**
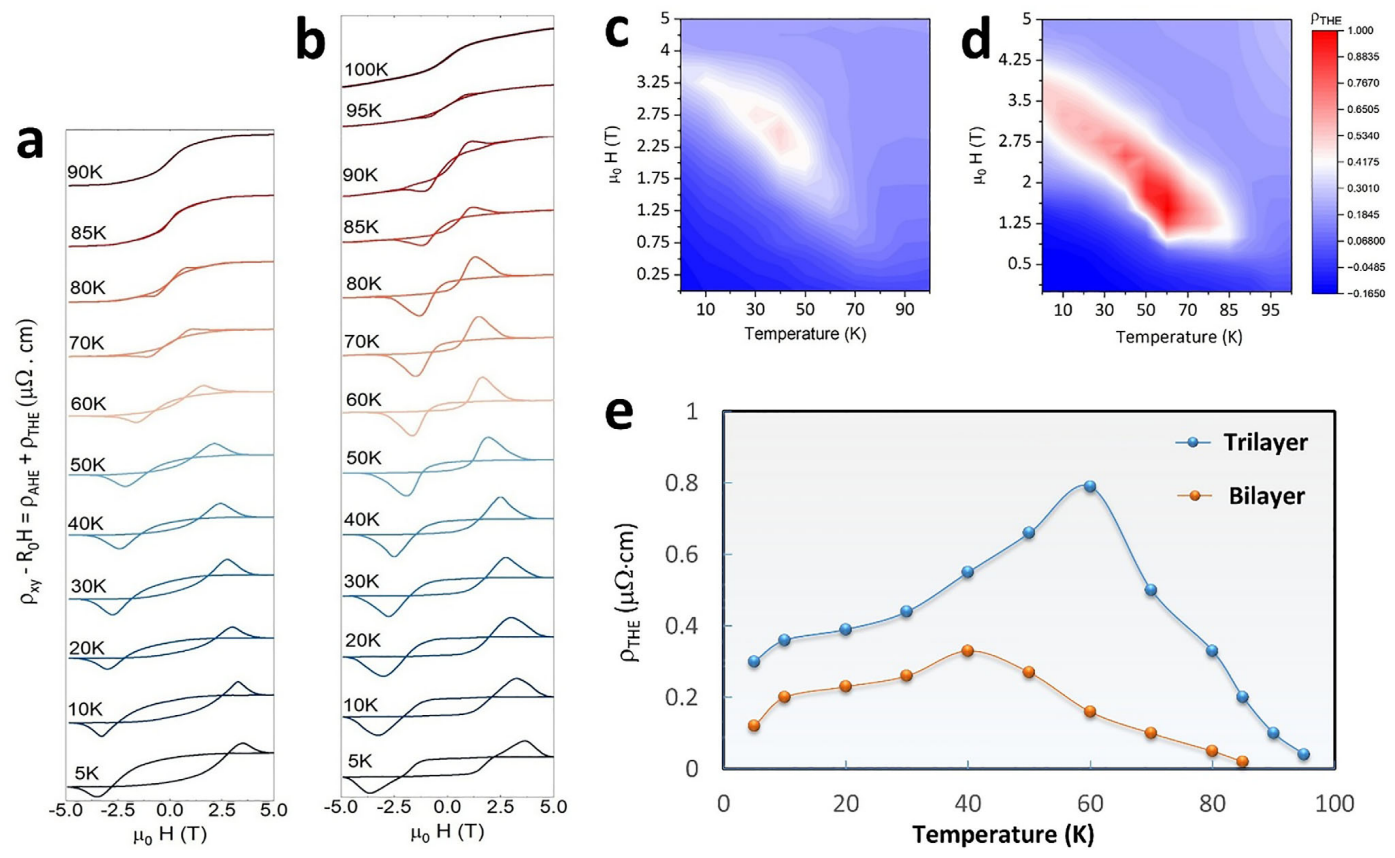
Topological‐Hall‐like response in the SRO/BTO bilayer and SRO/BTO/SRO trilayer. (a,b) Field‐dependent transverse Hall resistivity ρ_*xy*_(*H*)measured at different temperatures for the (a) bilayer BTO(10 u.c.)/SRO(4 u.c.)/STO and (b) trilayer SRO(4 u.c.)/BTO(10 u.c.)/SRO(4 u.c.)/STO. (c, d) Color maps of the extracted topological‐Hall‐like resistivity ρ_THE_ in the T‐µ_o_H plane for (c) the bilayer and (d) the trilayer. (e) Temperature dependence of ρ_THE_ for the bilayer and trilayer, showing a stronger and broader topological Hall response in the trilayer.

Phenomenologically, the measured Hall resistivity can be decomposed as: [7, 10, 15, 16, 17, 41]

(3) $$\rho_{xy}\left(H,T\right)=R_{0}\;\mu_{0}H+R_{s}M\left(H,T\right)+\rho_{\mathrm{THE}}\left(H,T\right)$$

where the first term is the ordinary Hall effect (OHE), the second term is the anomalous Hall effect (AHE), and the third term is the topological Hall contribution arising from a real‐space Berry phase associated with non‐coplanar spin textures. This form is widely used for SRO‐based systems showing topological Hall anomalies, including SRO/BTO, SRO/SIO, ultrathin SRO, and defect‐engineered SRO films [7, 10, 15, 16, 17]. We note that hump‐like Hall anomalies in oxide heterostructures can arise from multiple microscopic mechanisms, including noncoplanar magnetic textures, multichannel anomalous Hall effects, or magnetic inhomogeneity. Therefore, throughout this work, we conservatively describe the extracted contribution as a topological‐Hall‐like signal rather than definitive evidence consistent with noncoplanar magnetic textures. Nevertheless, the systematic enhancement observed specifically in the trilayer architecture strongly suggests that interfacial asymmetry and structural modulation play an important role in the enhanced topological‐Hall‐like response.

The field‐dependent *ρ_XY_* curves in Figure 2a,b reveal that both heterostructures exhibit an anomalous Hall background characteristic of ferromagnetic SRO, but the superposed hump‐like Hall anomaly is substantially more pronounced in the trilayer. This immediately indicates that the trilayer architecture does not merely preserve the interfacial chiral‐transport‐related Hall response found in the bilayer, but substantially enhances its magnitude and field‐temperature extent. The contour plots in Figure 2c,d make this distinction even clearer by showing that the topological signal occupies a wider and more intense region in the T‐H phase space for the trilayer than for the bilayer. The temperature dependence shown in Figure 2e further confirms that the trilayer sustains a larger ρ_THE_ over almost the entire measured range, with the strongest enhancement occurring in the low‐ and intermediate‐temperature regimes, where chiral spin textures are expected to coexist with robust ferromagnetic order.

This behavior is qualitatively consistent with previous reports on interfacial and ultrathin SRO systems. Matsuno et al., showed that in SRO/SIO bilayers, the Hall anomaly emerges as an additional contribution superimposed on the AHE, and that the extracted ρ_THE_ occupies a finite region in the *T*‐*H* plane, signaling the stabilization of a chiral magnetic phase [7]. They further emphasized that the topological component appears when the magnetic state begins to reverse from the saturated ferromagnetic configuration, indicating the coexistence of ferromagnetic order and a finite scalar spin chirality state. Similar behavior was later reported in ultrathin SRO single layers and defect‐engineered SRO, where hump‐like Hall anomalies persisted over broad temperature windows and were linked to inversion‐symmetry breaking, the DMI, and nontrivial spin textures. Our bilayer and trilayer data follow this general phenomenology; however, the magnitude and field–temperature persistence of the extracted hump‐like Hall contribution are substantially greater in the trilayer.

The residual hump‐like Hall component exhibits a substantially greater magnitude and a broader field–temperature distribution in the trilayer than in the bilayer. This systematic architecture‐dependent difference is the principal experimental result of Figure 2. However, the microscopic origin of the residual Hall contribution cannot be uniquely identified from transport measurements alone.

This ambiguity is particularly relevant to the present trilayer because both ultrathin SRO layers may contribute to the transverse transport response. The upper and lower SRO layers experience different structural and electrostatic boundary conditions and may therefore possess different anomalous Hall coefficients, carrier densities, magnetic anisotropies, coercive fields, and magnetization‐reversal pathways. The superposition of anomalous Hall contributions from magnetically or electronically nonequivalent SRO regions can produce a hump‐like Hall feature, as previously demonstrated in SRO‐based systems containing multiple conducting or magnetic channels [28, 29, 30, 32]. In complex oxide heterostructures, similar transport features may also originate from multichannel anomalous Hall effects, magnetic inhomogeneity, momentum‐space Berry‐curvature effects, or band‐structure reconstruction effects [42, 43, 44, 45].

At the same time, interfacial polar displacement, local inversion‐symmetry breaking, octahedral distortion, and Dzyaloshinskii–Moriya interaction are established mechanisms capable of producing noncoplanar magnetic configurations in ultrathin SRO and ferroelectric/SRO heterostructures [7, 10, 15, 16, 22, 30, 32]. Therefore, the present residual Hall contribution is described as a topological‐Hall‐like response because it is phenomenologically consistent with such configurations, while remaining compatible with a multichannel anomalous Hall contribution.

The complementary structural results presented below provide important evidence that the enhanced trilayer response is associated with a genuine architecture‐dependent modification rather than secondary‐phase formation, extensive intermixing, or loss of epitaxial integrity. In particular, the strain and polarization/displacement analyses demonstrate that the two SRO regions are structurally and electrostatically nonequivalent. However, this nonequivalence does not itself distinguish the two possible microscopic interpretations: it may modify interfacial DMI and support noncoplanar magnetic configurations, but it may also generate distinct anomalous Hall and magnetic‐switching responses in the two SRO layers.

The conclusion from Figure 2 is consequently comparative rather than mechanism‐specific: under the same measurement and extraction framework, the SRO/BTO/SRO trilayer exhibits a substantially stronger and more thermally persistent hump‐like topological‐Hall‐like response than the BTO/SRO bilayer. The transport results establish the architecture‐dependent enhancement, whereas identification of its precise microscopic origin requires consideration of the complementary structural, magnetic, and simulation results presented in the following sections.

### 2.3 Structural Characterization and Local Strain‐State Analysis

Figure 3 combines x‐ray diffraction (XRD), reciprocal space mapping (RSM), and TEM‐based strain analysis to probe the lattice state over both global and local length scales. The XRD patterns of both the bilayer and trilayer show only the expected (00*l*)reflections of BTO, SRO, and the STO substrate, without any detectable impurity‐related peaks, confirming phase‐pure and highly oriented growth of the films. In both samples, the presence of BTO(001)/BTO(002) and SRO(001)/SRO(002) reflections, together with the substrate peaks of STO(001), STO(002), and STO(003), indicates that the heterostructures are c‐axis‐oriented and epitaxially integrated with the STO substrate. Thus, at the global structural level, both the bilayer and trilayer retain good crystalline quality.

**FIGURE 3:**
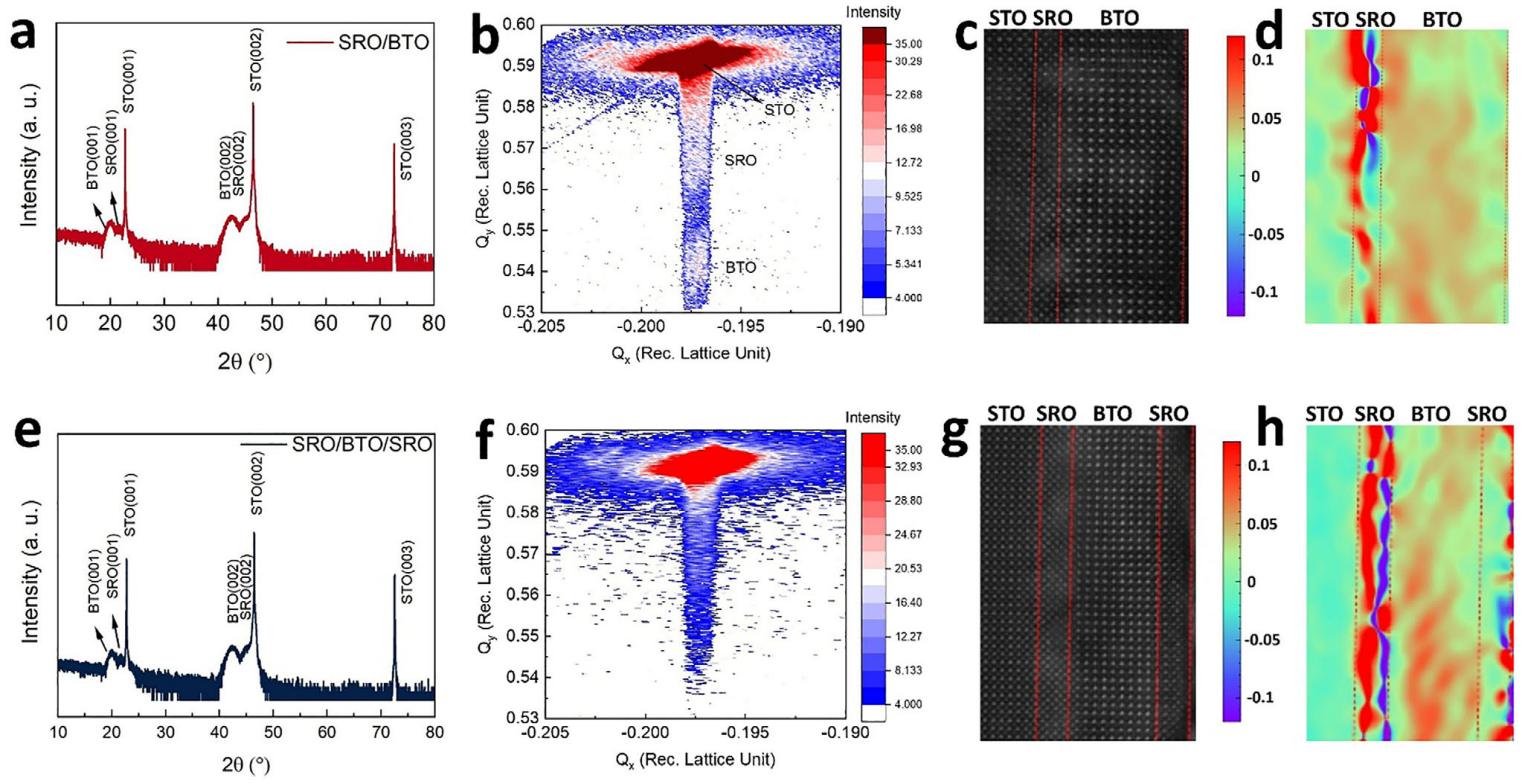
Structural characterization and local strain‐state analysis of bilayer and trilayer heterostructures. (a, e) XRD patterns of the bilayer BTO/SRO and trilayer SRO/BTO/SRO heterostructures, showing the expected (00*l*)reflections of BTO, SRO, and STO. (b, f) Reciprocal space maps of the bilayer and trilayer, respectively. The alignment of the film‐related scattering with the STO peak along *Q_x_* indicates that the oxide layers remained laterally locked to the substrate, which is consistent with coherent epitaxial growth. (c, d, g, h) TEM imaging and corresponding local strain maps of the bilayer and trilayer structures.

This was further supported by the RSM data. In both heterostructures, the film‐related scattering associated with BTO and SRO was aligned with the STO reflection along the Q*x* direction, indicating that the in‐plane lattice parameter of the oxide layers remained locked to that of the substrate. Such alignment is consistent with coherent epitaxial growth under strong in‐plane constraints from the STO. Simultaneously, the separation of the BTO‐ and SRO‐related features along Q_y_ reflects their different out‐of‐plane lattice spacings. The TEM strain contour plots in Figure 3 further reveal that the upper and lower SRO layers in the trilayer system are not structurally equivalent. The lower SRO layer exhibited alternating compressive and tensile domains, similar to the SRO layer in the bilayer, whereas the upper SRO layer appeared comparatively relaxed despite being epitaxially grown on a BTO layer. This structural picture is consistent with the supplementary microscopy and energy‐dispersive EDX analysis, which supports the integrity of the trilayer architecture (Figures S1 and S2).

The local strain is defined as

(4) $$\varepsilon =\frac{d_{\mathrm{local}}-d_{\mathrm{ref}}}{d_{\mathrm{ref}}}\;$$

where d_local_ is the measured lattice spacing, and d_ref_ is the corresponding reference spacing. In ultrathin SRO, such strain variations are closely coupled to octahedral distortion and local inversion‐symmetry breaking, both of which are known to influence the effective Dzyaloshinskii–Moriya interaction (DMI) and topological‐Hall‐like response [7, 10, 15].

This distinction is physically reasonable for SRO/BTO/SRO heterostructures. The lower SRO layer is directly constrained by both the STO and BTO layers, whereas the upper SRO layer, deposited on BTO, experiences a different elastic boundary condition. Previous studies on SRO/BTO/SRO capacitors have shown that local interface structure and termination can strongly influence the structural and ferroelectric response of the sandwiched BTO layer, rendering the upper and lower interfaces nonequivalent [27]. In the present case, the more relaxed state of the upper SRO layer indicates that the trilayer is not simply a duplicated bilayer but rather a vertically asymmetric heterostructure composed of two magnetically active SRO layers under different structural constraints.

The magnetic consequence of this asymmetry can be described through the magnetoelastic energy,

(5) $$\begin{array}{ccc} E_{\mathrm{me}} & = & B_{1}\;\left(\varepsilon_{xx}\alpha_{x}^{2}+\varepsilon_{yy}\alpha_{y}^{2}+\varepsilon_{zz}\alpha_{z}^{2}\right)\\ & & +\;2B_{2}\left(\varepsilon_{xy}\alpha_{x}\alpha_{y}+\varepsilon_{yz}\alpha_{y}\alpha_{z}+\varepsilon_{zx}\alpha_{z}\alpha_{x}\right) \end{array}$$

where B_1_ and B_2_ are the magnetoelastic coefficients, and α_i_ is the direction cosine of magnetization. In ultrathin SRO, strain is also coupled to cation–oxygen rumpling and octahedral tilting, so that local structural asymmetry can modify both the anisotropy and the interfacial DMI [10, 15, 16]. Therefore, the nonequivalent strain states of the upper and lower SRO layers are expected to produce different local magnetic environments within the trilayer, providing a plausible structural basis for the enhanced topological‐Hall‐like response.

The strain asymmetry also leads to asymmetry in the polar state, as shown in the polarization vector mapping in Figure 4. In both the bilayer and trilayer samples, the lower SRO region exhibited a non‐polar feature, as indicated by the limited magnitude of the polarization vector. In contrast, the upper SRO region in the trilayer exhibited a larger magnitude of the polarization vectors with a broader distribution of polarization orientations. These observations suggest that the upper and lower SRO/BTO interfaces in the trilayer are not electrostatically equivalent.

**FIGURE 4:**
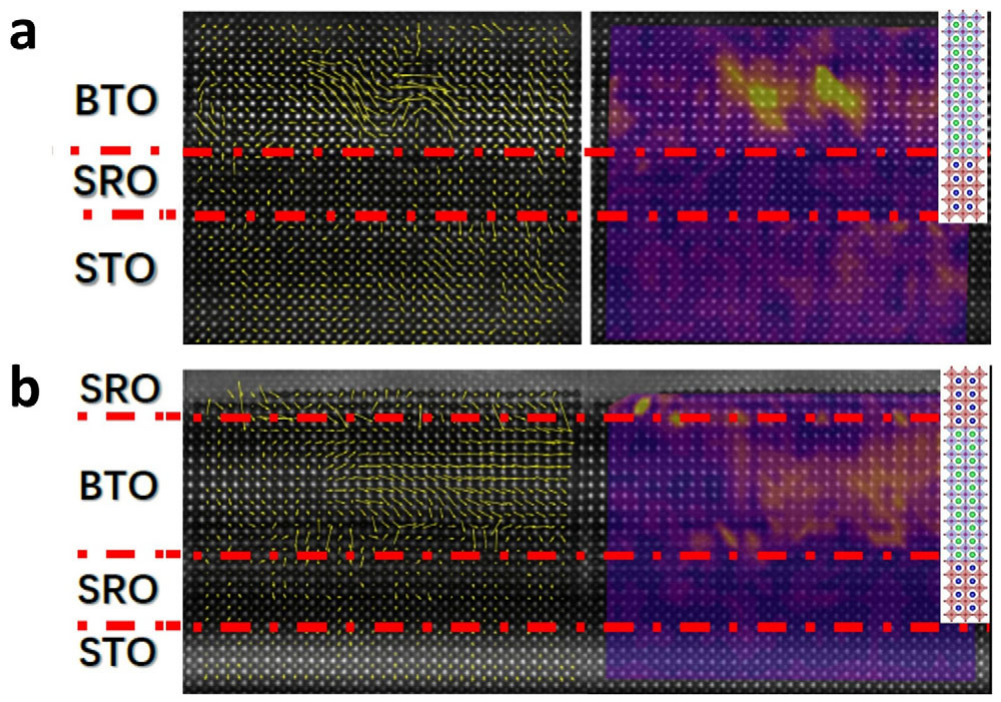
TEM‐based polarization‐vector and polarization‐magnitude mapping of the bilayer and trilayer heterostructures. (a) Cross‐sectional atomic‐resolution TEM image of the BTO(10 u.c.)/SRO(4 u.c.)/STO bilayer with overlaid local polarization/displacement vectors, together with the corresponding color‐coded polarization‐magnitude map shown in the right panel. (b) Cross‐sectional atomic‐resolution TEM image of the SRO(4 u.c.)/BTO(10 u.c.)/SRO(4 u.c.)/STO trilayer with overlaid local polarization/displacement vectors, together with the corresponding color‐coded polarization‐magnitude map shown in the right panel. Red dashed lines indicate the approximate layer interfaces. Yellow arrows represent the direction of local atomic displacement/polarization, while the color contrast in the right panels shows the relative magnitude and spatial distribution of the local polar distortion.

At the atomic scale, the spontaneous polarization can be related to the relative displacement of cations and anions within the perovskite unit cell,

(6) $$P_{s}=\frac{1}{\Omega }\;\sum_{i}Z_{i}^{*}\Delta u_{i},$$

where Ω is the unit cell volume, $Z_{i}^{*}$ is the Born effective charge, and Δu_i_ is the displacement of the ion *i*from its centrosymmetric position. In atomically resolved STEM analyses, the local polarization is often estimated from the relative displacement between the B‐site cation and the center of the surrounding A‐site framework, δ_A−B_, so that Ps ∝ δ_A−B_ [10, 33, 46].

This interpretation is also consistent with prior BTO/SRO studies, where ferroelectric distortion in BTO was shown to penetrate into interfacial SRO and generate a finite interfacial cation displacement, thereby enhancing inversion‐symmetry breaking and DMI [10]. Accordingly, a spatially nonuniform polar state in BTO is expected to produce a spatially nonuniform interfacial distortion landscape in adjacent SRO layers. The present data do not prove the formation of skyrmions by themselves, but they do show that the trilayer hosts a more complex polar environment than the bilayer. Together with the strain analysis in Figure 3, Figure 4 shows that the trilayer contains two structurally and electrostatically nonequivalent SRO environments. This vertical asymmetry provides a plausible architecture‐level basis for modifying the local magnetic energy landscape relative to the bilayer and may therefore contribute to the enhanced topological‐Hall‐like response.

Atomic‐resolution STEM and EDX results in Figures S2 and S3 confirm the intended SRO/BTO/SRO stacking sequence, chemically distinguishable layer boundaries, and limited elemental interdiffusion. The RHEED patterns and intensity oscillations in Figures S4 and S5 further demonstrate layer‐by‐layer epitaxial growth. Together with the phase‐pure XRD patterns, these results make secondary‐phase formation, extensive chemical intermixing, or loss of epitaxial integrity unlikely explanations for the enhanced trilayer Hall anomaly.

### 2.4 Surface Morphology and Its Possible Effect on the Topological Hall Response

Figure 5 compares the AFM topography of the bilayer and trilayer heterostructures and provides an additional structural correlate of the enhanced Hall anomaly and the enhanced topological‐Hall‐like response. The bilayer exhibits a low surface roughness, with Ra = 0.26 nm, whereas the trilayer is markedly rougher, with Ra = 0.9 nm. This large morphological difference is significant because the top SRO layer is only 4 u.c. thick, corresponding to a nominal thickness of 1.6 nm. The mean roughness of the trilayer is therefore already comparable to the full thickness of the top SRO layer, while the RMS roughness exceeds it. Such a result indicates that the trilayer cannot be regarded as an atomically flat and laterally uniform magnetic overlayer; instead, it should be viewed as a structurally modulated ultrathin SRO layer grown on a ferroelectric BaTiO_3_ spacer. This point is particularly relevant because Figure 5 is explicitly the AFM comparison between bilayer and trilayer samples, while the preceding figures already show that the trilayer also possesses a nonequivalent upper SRO layer in both strain and polarization environments.

**FIGURE 5:**
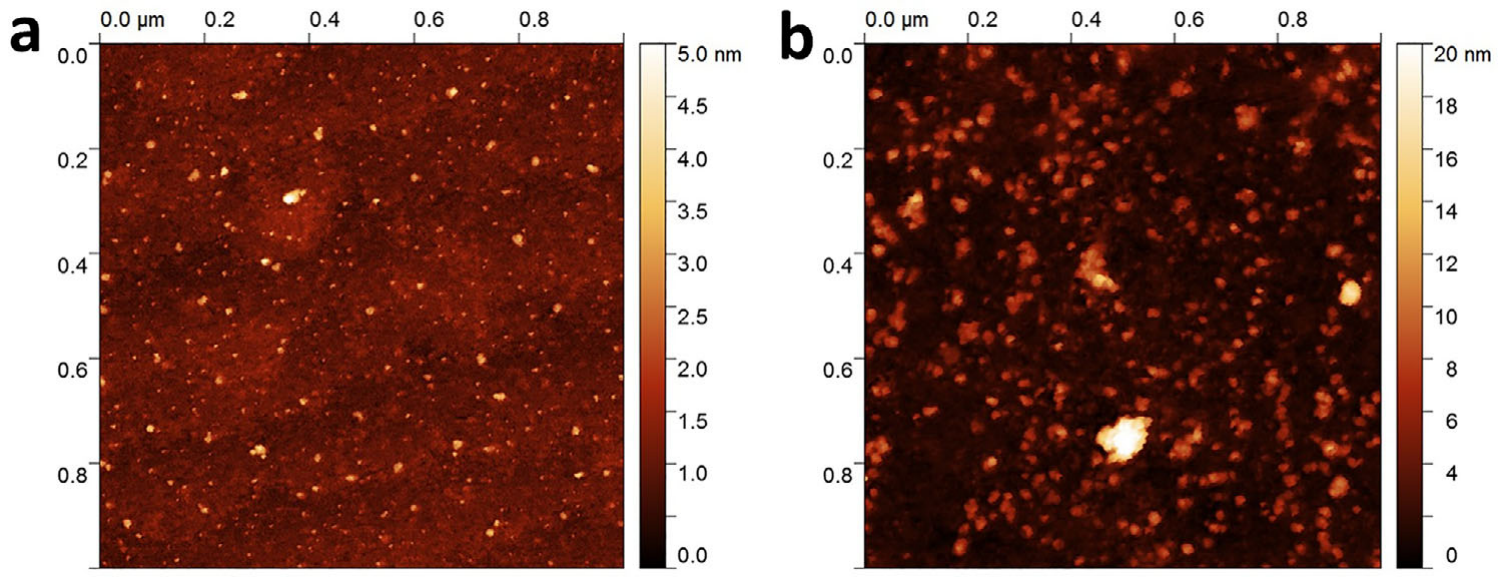
AFM topography of the bilayer and trilayer heterostructures. (a) AFM image of the bilayer BTO(10 u.c.)/SRO(4 u.c.)/STO. (b) AFM image of the trilayer SRO(4 u.c.)/BTO(10 u.c.)/SRO(4 u.c.)/STO.

The roughness difference may contribute to the experimentally observed enhancement of the topological‐Hall‐like response in the trilayer. In an idealized model, adding a second SRO layer to form SRO/BTO/SRO would not necessarily guarantee a dramatic enhancement of THE. If both SRO/BTO interfaces were atomically flat, laterally uniform, and magnetically equivalent, then the trilayer might be expected to show only a modest quantitative change relative to the bilayer. However, the present heterostructure is clearly not such an ideal symmetric stack. Figures 3 and 4 already indicate that the lower and upper SRO layers in the trilayer are structurally inequivalent: the lower SRO is substrate‐clamped, whereas the upper SRO is more relaxed and experiences a more disordered polarization environment. The AFM data now show that the top surface of the trilayer is also far more corrugated than that of the bilayer. Taken together, these results indicate that the upper SRO layer in the trilayer is not simply an additional ferromagnetic layer, but a structurally and electrostatically nonuniform magnetic environment.

In ultrathin oxide ferromagnets, such roughness is expected to locally influence the magnetic energy balance through structural modulation, i.e.,

(7) $$\begin{array}{ccc} E\left(r\right) & = & A\left(r\right){\left(\nabla m\right)}^{2}+D\left(r\right)\left[m_{z}\left(m\cdot \nabla \right)-\left(\nabla \cdot m\right)m_{z}\right]\\ & & -\;K_{\mathrm{eff}}\left(r\right){\left(n\cdot m\right)}^{2}-\mu_{0}M_{s}\left(r\right)H\cdot m+E_{\mathrm{IEC}}\left(r\right) \end{array}$$

where *A*, *D*, *K_eff_*, and the effective indirect magnetostatic/strain/polarization‐mediated influence coupling E_IEC_ are all allowed to be laterally nonuniform. In the trilayer, the very large increase in roughness implies that the upper SRO layer contains strong local variations in thickness, bonding geometry, screening condition, and octahedral connectivity. Since the SRO is only 4 u.c. thick, these variations are not minor perturbations. Rather, they can substantially modify the local balance between ferromagnetic exchange and interface‐driven DMI. The local threshold for formation of a chiral texture may be written as

(8) $$D_{c}\left(r\right)=\frac{4\sqrt{A\left(r\right)K_{\mathrm{eff}}\left(r\right)}}{\pi }\;$$

so that the roughness‐induced structural modulation may locally alter the balance between exchange interaction, anisotropy, and interfacial DMI, where D(**r**)≥D_c_(**r**), even if the spatially averaged structure would not suggest a large enhancement.

This interpretation is consistent with the broader behavior of SRO‐based oxide systems. In BTO/SRO heterostructures, ferroelectric distortion in BTO induces ionic displacement in adjacent SRO and thereby generates interfacial DMI [10]. In ultrathin SRO films, interfacial oxygen‐octahedral tilting and structural asymmetry are likewise known to promote THE by breaking inversion symmetry [16]. Qin et al., further showed that even in nominally single‐layer SRO, topological Hall transport is strongly tied to surface/interface effects, underscoring the sensitivity of ultrathin SRO to surficial structural modulation [15]. In addition, mixed or nonuniform interface termination in SRO/BTO/SRO capacitors has been shown to produce strong lateral variation in the ferroelectric response, including pinned dipoles and spatially inhomogeneous polarization [27]. The present AFM result therefore fits naturally with the preceding strain and polarization analyses: the rough trilayer topography is not an isolated geometric feature, but rather a manifestation of a laterally varying structural and electrostatic landscape that can strongly modulate the local DMI.

The roughness can enhance THE through several coupled routes. First, it generates local thickness modulation in the top SRO layer. This is particularly important because previous studies have shown that THE in oxide heterostructures is highly sensitive to ferromagnetic‐layer thickness, reflecting the competition between the interfacially distorted region and the undistorted interior of the magnetic layer [33]. In the present case, where the nominal top SRO thickness is only ∼ 1.6 nm, a roughness amplitude of 0.9 nm implies that the local electronic and magnetic state of the top SRO layer can vary strongly across the sample. Second, roughness is expected to modify the local Ru─O rumpling amplitude and hence the local DMI strength, since to first order

(9) $$|D\left(r\right)|\propto \delta_{\mathrm{Ru}-O}\left(r\right)$$

Third, the pronounced surface roughness introduces a strongly modulated lateral landscape. In ultrathin oxide layers, such topographic and structural variations can locally perturb magnetic exchange, anisotropy, and electrostatic screening. Rather than acting purely as detrimental disorder, this lateral structural modulation conceptually broadens the distribution of local magnetic interactions, which may contribute to the extended field and temperature stability of the observed topological transport.

This is also the point at which the AFM data connect most naturally with the micromagnetic results. The simulations in Figures 7 and 8 indicate that the trilayer is intrinsically favorable for stabilizing a chiral state because interfacial DMI acts together with indirect interlayer influence across the BTO spacer. The AFM data suggest why this mechanism becomes much stronger in the real trilayer than in an ideal flat trilayer. The top SRO layer is evidently much more nonuniform than the bottom layer, so the real structure contains a distribution of local D, K_eff_, and perhaps coupling efficiency, rather than a single set of uniform parameters. Some regions of the upper SRO layer will therefore act as preferential regions for stabilizing nonuniform chiral spin configurations, while the coupling to the lower SRO layer through BTO can help preserve these textures over a wider field and temperature window. In this sense, surface roughness should not be regarded as the sole origin of the enhanced topological Hall resistivity, ρ_THE_, but as a possible contributor to the broader distribution of local magnetic and transport environments of the interfacial DMI and indirect interlayer‐influence mechanism already identified from transport, TEM, and simulation.

Theory based on a laterally uniform trilayer may predict only a limited difference between bilayer and trilayer. Experimentally, however, the trilayer is not laterally uniform. It combines a second magnetic layer with a much rougher topography, a more relaxed upper SRO strain state, and a more disordered polarization environment. The result is a broader distribution of local magnetic interaction landscapes within the trilayer. This interpretation is also consistent with prior multiferroic oxide studies showing that ferroelectric distortion and indirect interlayer influence can extend interfacial topological states well beyond what would be expected from a purely single‐interface model [7, 33].

Taken together, the AFM results show that the trilayer is not only structurally different in layer sequence, but also markedly less uniform than the bilayer. Its much larger roughness, comparable to the thickness of the top SRO layer, suggests that the upper SRO acts as a structurally modulated chiral interface. Combined with the strain asymmetry, polarization disorder, and indirectly assisted stabilization inferred from micromagnetic simulations, this supports the conclusion that the enhanced topological‐Hall‐like resistivity, ρ_THE_, in the trilayer arises from the cooperative effects of interfacial DMI, vertical structural asymmetry, indirect interlayer influence, and roughness‐assisted stabilization of spatially nonuniform chiral spin configurations.

### 2.5 Magnetic and Longitudinal Transport Properties

Figure 6 provides the essential transport and magnetization background for interpreting the enhanced topological‐Hall‐like response of the trilayer. As summarized in the figure description, Figure 6a shows the temperature dependence of the longitudinal resistivity ρ_xx_(T), Figure 6b shows the out‐of‐plane magnetization measured at 0.05 T, and Figure 6c,d present the 4 K magnetization loops measured along the in‐plane and perpendicular directions for the bilayer and trilayer, respectively. Due to the extremely small magnetic signal from ultrathin SRO layers, the extracted absolute magnetic moment is highly sensitive to background subtraction and fitting procedures. Therefore, the magnetic analysis here focuses primarily on the relative evolution of the M‐H behavior rather than the absolute µ_B_/Ru values. Taken together, these measurements establish that both heterostructures remain conducting and ferromagnetic in the same temperature regime where the Hall anomalies are observed, and they provide the magnetic context needed to relate the transport response in Figure 2 to the interfacial structural distortions discussed earlier.

**FIGURE 6:**
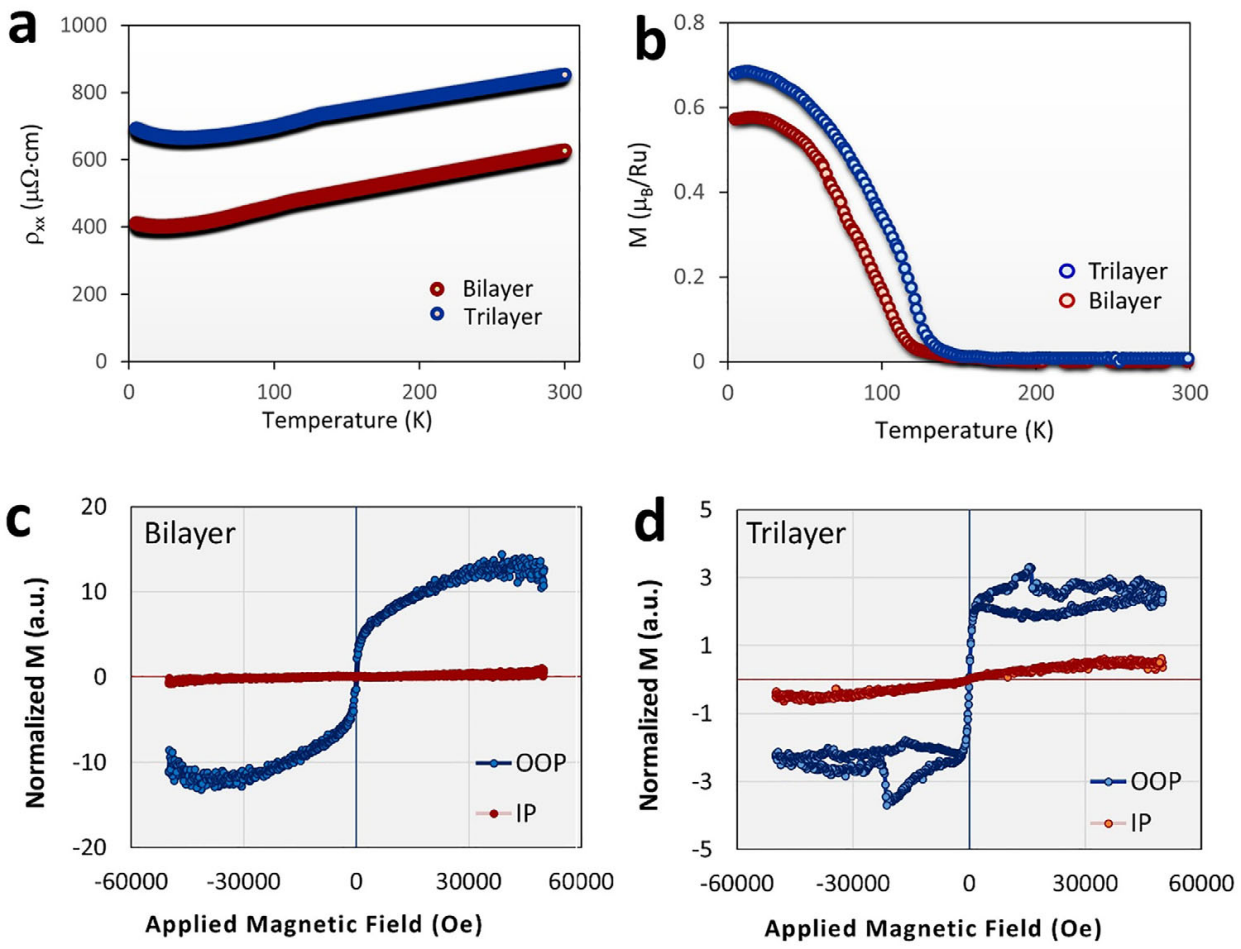
Magnetic and transport properties of the bilayer and trilayer heterostructures. (a) Temperature‐dependent longitudinal resistivity ρ_*xx*_(*T*)of the bilayer BTO(10 u.c.)/SRO(4 u.c.)/STO and the trilayer SRO(4 u.c.)/BTO(10 u.c.)/SRO(4 u.c.)/STO. (b) Temperature‐dependent out‐of‐plane magnetization measured at 0.05 T for the bilayer and trilayer. (c, d) Field‐dependent magnetization measured at 4 K with the magnetic field applied along the in‐plane and out‐of‐plane directions for the (c) bilayer and (d) trilayer.

The temperature dependence of the longitudinal resistivity may be written phenomenologically as

(10) $$\rho_{xx}\left(T\right)=\rho_{0}+\rho_{\mathrm{ph}}\left(T\right)+\rho_{\mathrm{sd}}\left(T\right)$$

where ρ_0_ is the residual resistivity, ρ_ph_ is the phonon‐scattering contribution, and ρ_sd_ is the spin disorder term. In itinerant ferromagnetic oxides such as SRO, ρ_sd_ is strongly suppressed below the onset of ferromagnetic order, so that the resistivity commonly deviates from its higher‐temperature behavior near the magnetic transition [10, 47]. Therefore, the ρ_xx_(T) and M(T) curves in Figure 6 should be considered together. Their correlated evolution indicates that the conducting state in both heterostructures is governed by the magnetic SRO layers, consistent with the general behavior reported for ultrathin SRO‐based films and oxide superlattices, where the magnetic transition leaves a clear signature in both transport and magnetization [15, 16, 47].

This is important for the interpretation of Figure 2. The larger topological Hall‐like signal in the trilayer cannot be attributed simply to the absence of ferromagnetism in the bilayer or to a trivial insulating transition in any of the samples. Figure 6 shows that both heterostructures support an underlying ferromagnetic conducting matrix, upon which the topological Hall‐like contribution is superposed. This is consistent with prior SRO‐based studies, where topological Hall anomalies develop on top of a conventional anomalous Hall background in films that remain ferromagnetic and sufficiently conducting [7, 15]. The enhanced Hall anomaly is observed within a ferromagnetic conducting background and is associated with an architecture‐dependent modification of the magnetic‐reversal and transverse‐transport landscape. However, the present measurements do not uniquely determine whether this modification arises from noncoplanar magnetism, multichannel anomalous Hall transport, or their coexistence.

The 4 K magnetization loops in Figure 6c,d indicate different magnetic anisotropy and reversal behavior between the bilayer and trilayer, consistent with the strain and polar asymmetry revealed by the structural analysis [15, 16, 27, 48].

Field‐dependent magnetization is also directly relevant to the extraction of the topological Hall contribution. In SRO‐based systems, the topological Hall signal typically appears in the field range where the magnetization begins to reverse from the saturated ferromagnetic state and vanishes again once the magnetization is fully aligned at higher field [7, 17, 33]. Matsuno et al., showed in SRO/SIO bilayers that the field at which THE reaches its maximum tracks the magnetic reversal regime, indicating the appearance of finite scalar spin chirality during the switching process [7]. Similar conclusions were reached for ultrathin SRO films and defect‐engineered SRO, where the hump‐like Hall anomaly emerges on top of an otherwise conventional ferromagnetic hysteresis loop [17, 33]. The M‐H measurements in Figure 6 therefore provide the magnetic baseline for the Hall analysis in Figure 2, confirming that the topological‐Hall‐like response develops within a ferromagnetic background rather than in a separate magnetic phase.

The M–H curves and Hall hysteresis loops should not be expected to coincide exactly in the central switching region. The M–H loop reflects the spatially averaged magnetic moment of the full, comparatively large‐area ultrathin heterostructure, whereas the Hall signal selectively probes the electrically conducting SRO channel within the patterned transport device and is sensitive to local reversal processes, anomalous Hall contributions, and possible noncoplanar interfacial spin configurations. In addition to these different measurement sensitivities, microfabrication of the Hall device may also influence the magnetic reversal process. Lithographic patterning and the associated etching or ion‐milling steps can introduce local strain relaxation, structural disorder, edge damage, and geometrical confinement in the ultrathin SRO channel, thereby creating additional domain‐wall pinning sites and shifting or broadening the effective coercive field. Patterned SRO nanostructures have previously been shown to exhibit pronounced geometry‐dependent reversal and strong domain‐wall pinning [49]. Therefore, part of the difference between the macroscopic M–H loop and the Hall hysteresis may originate from device fabrication. Because a direct before‐and‐after fabrication comparison was not performed, the magnitude of this contribution cannot be quantified here. The M–H data are consequently used primarily to establish the ferromagnetic background and the broad reversal regime, while the hump‐like Hall feature is treated as a comparative topological‐Hall‐like signature rather than standalone proof of a real‐space nontrivial magnetic texture.

These magnetic differences further support the view that the trilayer architecture modifies the magnetic energy landscape of ultrathin SRO. In ultrathin SRO, even modest changes in lattice distortion or interfacial bonding can alter the exchange interaction, the orbital character near the Fermi level, and the balance between anisotropy and DMI [10, 16, 27, 50]. Therefore, the additional SRO/BTO interface in the trilayer is expected to modify the polar distortion profile and influence the magnetic stiffness and reversal pathway of the system. Figure 6 shows the enhanced topological‐Hall‐like response of the trilayer within this broader magnetic framework. After subtraction of the linear diamagnetic substrate contribution, the saturation magnetic moment of the trilayer was estimated to be approximately 1.5–2.0 µ_*B*_/Ru at 4 K, consistent with previously reported values for ultrathin SrRuO_3_ films and heterostructures.

### 2.6 Micromagnetic Simulations of Monolayer, Bilayer and Trilayer Architectures

To examine how magnetic architecture affects the stability of noncoplanar chiral magnetic configurations, we performed qualitative micromagnetic simulations for SRO‐based model systems, including a 4‐u.c. SRO monolayer, a BTO/SRO bilayer, and an SRO/BTO/SRO trilayer. The monolayer simulations were used as a baseline to test relaxation from different initial magnetic states and to validate the selected DMI–anisotropy parameter range, as shown in Figures S6–S8. The bilayer and trilayer simulations were then compared under the same DMI range and initial magnetic conditions in Figure 7, while Figure 8 and Figure S9 further examine the robustness of the trilayer configuration. Therefore, the simulations should be regarded as a qualitative comparison of architecture‐dependent magnetic stability rather than a quantitative reconstruction of the exact experimental spin texture.

**FIGURE 7:**
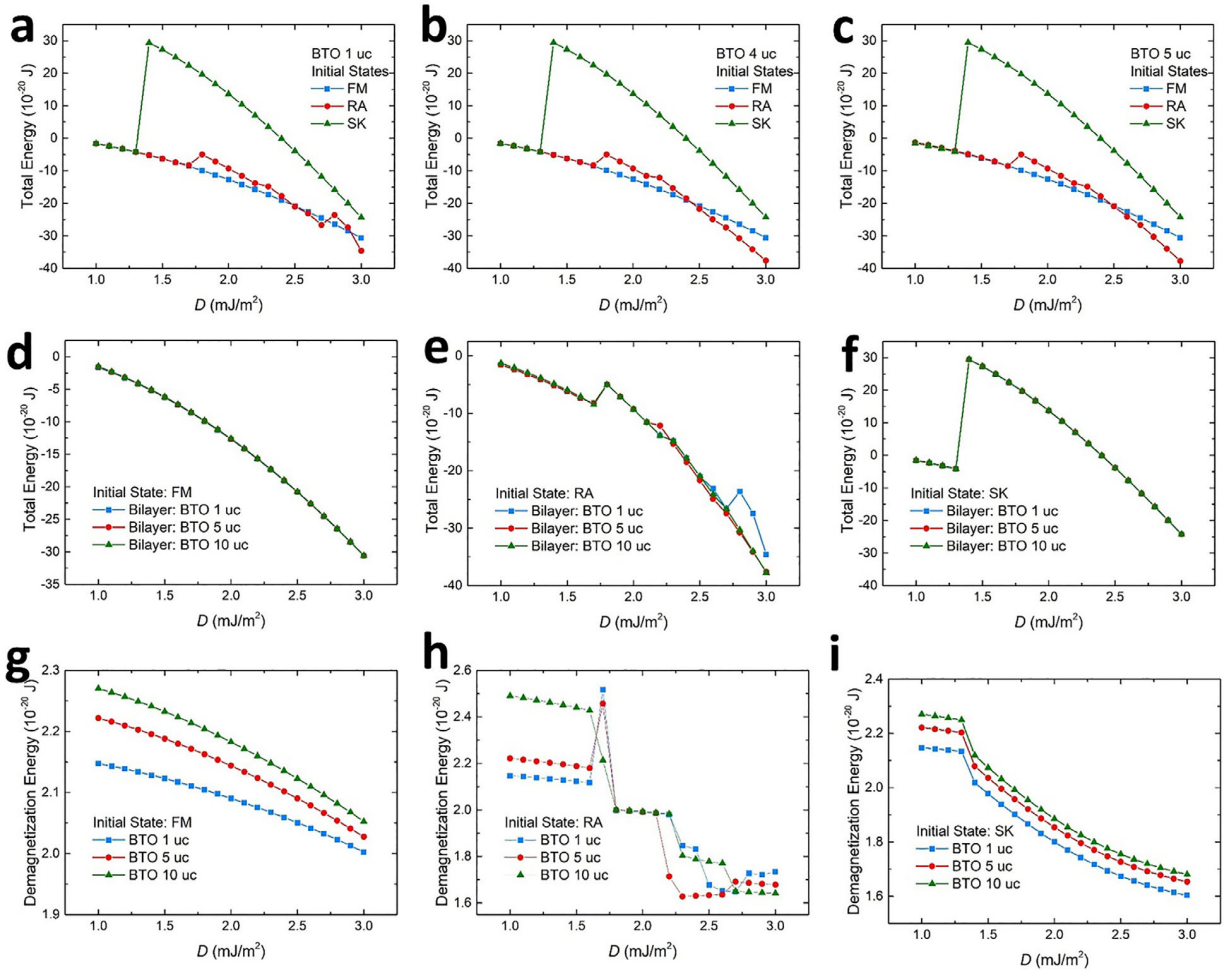
Micromagnetic comparison of bilayer and trilayer architectures under varied DMI strength and initial magnetic states. (a–c) Total energy as a function of DMI strength D for the SRO/BTO/SRO trilayer with BTO spacer thicknesses of 1, 4, and 5 u.c., respectively, using ferromagnetic (FM), random (RA), and skyrmion‐like (SK) initial states. (d–f) Corresponding total energy for the BTO/SRO bilayer with different BTO thicknesses under the same FM, RA, and SK initial states. (g–i) Corresponding demagnetization energy as a function of D for the same simulated conditions. The comparison is used to evaluate how the magnetic‐energy landscape changes when the architecture is modified from bilayer to trilayer.

**FIGURE 8:**
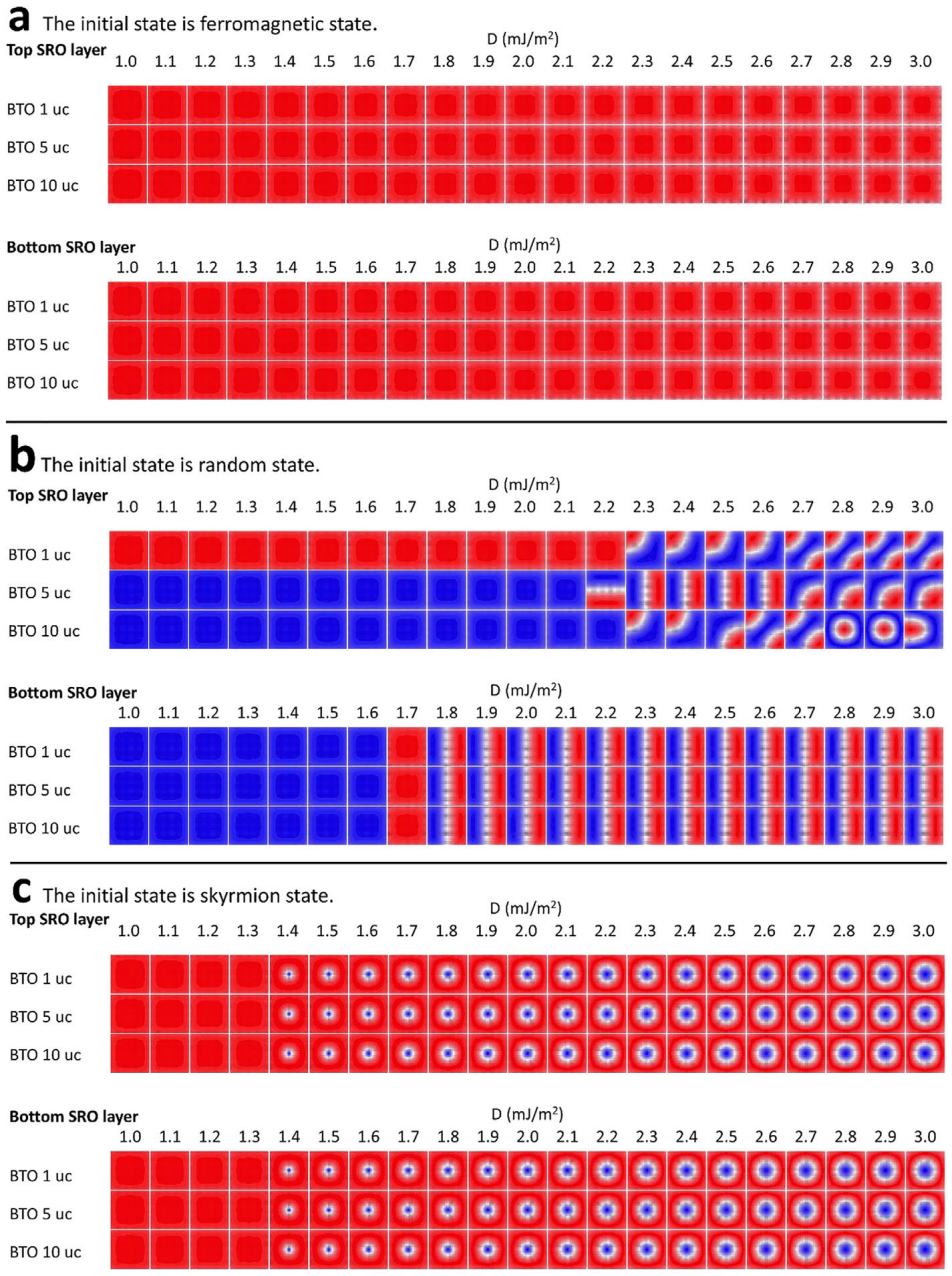
Robustness of the simulated chiral state in the SRO/BTO/SRO trilayer. Micromagnetic results for the trilayer using *A* = 5 pJ m^−1^, *D* = 1.0−3.0 mJ m^−2^, *Ms* = 0.19 MA m^−1^, *K* = 0.5 MJ m^−3^ for SRO, with the BTO spacer treated as a nonmagnetic layer.

The time evolution of the magnetization is governed by the Landau–Lifshitz–Gilbert (LLG) equation,

(11) $$\frac{\partial m}{\partial t}=-\gamma_{0}\;m\times H_{\mathrm{eff}}+\alpha \left(m\times \frac{\partial m}{\partial t}\right)$$

where **m** is the unit magnetization vector, *γ*_0_ is the absolute ratio, α is the Gilbert damping constant, and H_eff_ is the effective magnetic field derived from the total free energy. For the present trilayer, the magnetic energy density can be written as

(12) $$\begin{array}{ccc} E & = & A{\left(\nabla m\right)}^{2}+D\left[m_{z}\left(m\cdot \nabla \right)-\left(\nabla \cdot m\right)m_{z}\right]\\ & & -\;K{\left(n\cdot m\right)}^{2}-\mu_{0}M_{s}m\cdot H+E_{\mathrm{dip}}+E_{\mathrm{IEC}} \end{array}$$

where the first four terms represent the ferromagnetic exchange interaction, DMI, uniaxial anisotropy, and Zeeman contributions, respectively, E_dip_ denotes the demagnetization energy, and E_IEC_ represents a phenomenological indirect interlayer term accounting for non‐exchange coupling effects between the two SRO layers, including magnetostatic/dipolar interaction and possible strain‐ or polarization‐mediated boundary effects through the BTO spacer. This form follows the standard micromagnetic description of interfacial skyrmion systems and is particularly relevant here because the trilayer differs from the bilayer not only by the presence of an additional magnetic layer, but also by the possibility of indirect interlayer influence across the ferroelectric spacer [7, 10, 15, 17].

Figure 7 compares the simulated energy evolution of the bilayer and trilayer geometries under the same DMI range and initial magnetic conditions. The comparison indicates that the trilayer architecture modifies the magnetic‐energy landscape relative to the bilayer and remains compatible with noncollinear or skyrmion‐like relaxed configurations over a finite DMI range. This trend is qualitatively consistent with the experimentally observed enhancement of the topological‐Hall‐like response in the trilayer. However, the simulations should not be interpreted as direct proof of skyrmions; rather, they provide qualitative support that the trilayer geometry can offer a favorable magnetic environment for noncoplanar chiral configurations.

Within this framework, the simulations provide qualitative support that the two‐SRO‐layer trilayer geometry modifies the magnetic‐energy landscape relative to the single‐SRO‐layer/bilayer model and remains compatible with noncoplanar or chiral spin configurations over a finite range of DMI and anisotropy parameters [7, 10, 33, 46, 50]. The experimentally observed strain and polarization asymmetry between the upper and lower SRO layers may further broaden the local distribution of magnetic parameters. These simulations should therefore be interpreted as qualitative support for the proposed mechanism, rather than direct evidence of skyrmions or a quantitative prediction of the experimental spin texture [51].

Figure 8 further examines the robustness of the simulated trilayer magnetic configuration. In contrast to Figures S6–S8, which provide monolayer baseline simulations, Figure 8 focuses on the SRO/BTO/SRO trilayer and shows how the relaxed magnetic state evolves with DMI strength, BTO spacer thickness, and initial magnetic condition [52]. The corresponding anisotropy–DMI parameter sweep for the trilayer is shown in Figure S9. These results support the view that the trilayer configuration remains compatible with chiral magnetic states over a finite parameter range, but they are not intended as standalone proof of the microscopic spin texture.

Likewise, the anisotropy sweep in Figure S9 shows that the chiral state persists over a finite range of *K*rather than collapsing immediately under modest parameter variation. This behavior is physically consistent with the experimental heterostructure, since the strain maps and polarization analysis already imply that K, D, and possibly the effective interlayer coupling are distributed across the film rather than perfectly uniform.

A central quantity for characterizing the simulated texture is the topological charge [53],

(13) $$Q=\frac{1}{4\pi }\;\int m\cdot \left(\frac{\partial m}{\partial x}\times \frac{\partial m}{\partial y}\right)\;dx\;dy$$

where m is the normalized magnetization. Although the present simulations are qualitative and do not provide an experimental measurement of skyrmion number, the persistence of nontrivial simulated textures over finite ranges of **D** and **K** supports the view that the trilayer geometry is more favorable for noncoplanar magnetic states than the bilayer [16].

The inclusion of the BTO spacer as a nonmagnetic layer with zero saturation magnetization is also physically meaningful. It emphasizes that the BTO layer does not contribute a direct ferromagnetic moment, but modifies the magnetic texture of the adjacent SRO layers through interfacial symmetry breaking, ionic displacement, and the coupling pathway between the two SRO layers. This picture is well aligned with prior studies on BTO/SRO heterostructures, where ferroelectric distortion in BTO was shown to induce interfacial cation displacement in SRO and thereby enhance DMI [10], and with multiferroic FM/ferroelectric/AFM systems, where an additional exchange channel substantially enlarges the phase space for stabilization of noncollinear chiral spin textures. In that sense, the present trilayer differs fundamentally from a single SRO layer or a single‐interface bilayer: the spacer is not magnetically active by itself, but it reshapes the magnetic energy landscape on both sides of the interface.

The robustness seen in Figure 8 is important for interpreting the transport data. In oxide systems, hump‐like Hall anomalies can in some cases arise from superposed AHE contributions or band‐structure reconstruction rather than a genuine topological‐Hall‐like response [9]. A simulation result that survives multiple initial states and parameter sweeps does not by itself prove the presence of skyrmions, but it substantially strengthens the case that the trilayer geometry is intrinsically favorable for a noncoplanar chiral magnetic state. This is particularly true here because the parameter robustness in Figure 8 is consistent with the independent structural evidence for nonequivalent top and bottom SRO layers and with the experimentally broadened—window of THE in the trilayer.

## 3 Conclusions

In summary, conversion of the BTO/SRO bilayer into an SRO/BTO/SRO trilayer produces a markedly stronger and broader topological‐Hall‐like response, with a maximum extracted residual Hall resistivity of approximately 0.8 µΩ cm and a temperature window extending from 5 to 90 K. This architecture‐dependent enhancement is the central experimentally established result of the present work.

XRD, RSM, atomic‐resolution TEM, strain mapping, and polarization/displacement analysis demonstrate that the trilayer retains high structural quality while containing nonequivalent upper and lower SRO environments. The lower SRO layer remains comparatively substrate‐constrained, whereas the upper SRO layer is more relaxed and exhibits a spatially more heterogeneous polar environment. AFM further reveals substantially enhanced morphological modulation in the trilayer. Micromagnetic simulations demonstrate that the trilayer architecture can support noncoplanar relaxed magnetic configurations over a finite parameter range.

Nevertheless, the structural and electrostatic nonequivalence of the two SRO layers may also generate different anomalous Hall coefficients, coercive fields, and magnetic‐reversal pathways, allowing a multichannel AHE contribution to the hump‐like Hall response. The present results therefore do not uniquely assign the anomaly to a single microscopic mechanism. Instead, they demonstrate that trilayer architecture strongly modifies the structural, magnetic, and transverse‐transport landscape of ultrathin SRO and produces a significantly stronger and more persistent topological‐Hall‐like response than the corresponding bilayer. The combined results remain physically consistent with a contribution from noncoplanar magnetism, while allowing for the possible coexistence of multichannel anomalous Hall effects.

## 4 Methods and Materials

The bilayer and trilayer thin films are composed of two perovskite layers with different thickness sandwiched between electrodes, which are in series with each layer as schematically illustrated in Figure S1. Epitaxial BTO(10 u.c.)/SRO(4 u.c.) bilayer and SRO(4 u.c.)/BTO(10 u.c.)/SRO(4 u.c.) trilayer thin films were deposited on SrTiO3 (STO) (001) single‐crystal substrates by laser molecular beam epitaxy (LMBE). A KrF excimer laser (248 nm) with an energy density of 2 and 1.2 J/cm^2^ was adopted for the deposition of SRO and BTO layers, respectively. The growth of BTO and SRO layers was carried out at a substrate temperature of 700°C, respectively, under an oxygen partial pressure of 10 mTorr using ceramic targets of respective materials. The repetition rate of the laser was kept at 3 Hz, and the distance between the targets and substrates was fixed at 5 cm

The crystallographic structure was characterized by X‐ray diffraction (Rigaku, 9 kW rotating anode thin‐film XRD). The cross‐sectional interfacial structure was observed by an aberration‐corrected JEOL GARM transmission electron microscopy (ACTEM) at 300 kV with a double corrector with a convergence angle of 67 mrad. Images were acquired using a standard set of atomic‐resolution high‐angle annular dark field (HAADF) STEM conditions. Here, we use a 30 µm condenser aperture, a probe current of 105 pA (300 kV) for the HAADF STEM imaging. The cross‐sectional TEM specimens were prepared using focused ion beam milling, followed by thinning with Ar ion milling. The same TEM instrument with a point resolution of less than 0.08 nm was used for the energy‐dispersive x‐ray spectroscopy (EDS) element mapping. The ACTEM is equipped with a single 100mm^2^ silicon drift x‐ray detector (JED‐2300T) for EDX acquisition.

Magnetic properties were characterized using a Physical Property Measurement System equipped with a Vibrating Sample Magnetometer (PPMS‐VSM). The external magnetic field was applied both parallel (in‐plane) and perpendicular (out‐of‐plane) to the SrTiO_3_ (001) substrate to probe magnetic anisotropy. Prior to measurement, the samples were cooled from room temperature to 5 K under an applied magnetic field of 1 kOe (field‐cooled condition). Magnetic hysteresis (M–H) loops were subsequently recorded at 4 K by sweeping the magnetic field between −50 kOe and +50 kOe. To isolate the intrinsic ferromagnetic contribution of the ultrathin SRO layers, the raw magnetization data were corrected by subtracting the linear diamagnetic background associated primarily with the STO substrate. The background slope was determined from the high‐field region where the magnetization approached saturation.

The transverse Hall resistivity was analyzed using the standard decomposition

(14) $$\begin{array}{ccc} \rho_{xy}\left(H\right) & = & R_{0}\;\mu_{0}H+\rho_{\mathrm{AHE}}\left(H\right)+\rho_{\mathrm{THE}}\left(H\right)=R_{0}\;\mu_{0}H+R_{s}M\left(H\right)\\ & & +\rho_{\mathrm{THE}}\left(H\right) \end{array}$$

where *R*_0_µ_0_ *H*, ρ_AHE_, and ρ_THE_ denote the ordinary, anomalous, and topological Hall contributions, respectively [7, 10, 15, 16]. Following the procedure commonly adopted for SRO‐based topological Hall systems, the ordinary Hall background was first subtracted by linear fitting of the high‐field region, where the Hall response is dominated by the ordinary term [7, 10, 20]. The anomalous Hall contribution was then determined from the field‐dependent magnetization, using the high‐field saturated regime in which the spins are ferromagnetically aligned and the topological contribution is expected to vanish [7, 15, 16]. The residual hump‐like component obtained after subtraction of the ordinary and anomalous Hall terms was assigned to ρ_THE_, consistent with previous analyses of SRO/BTO, SRO/SrIrO_3_, and ultrathin SRO systems [7, 10, 15, 16].

### 4.1 Micromagnetic Simulation Methods

In micromagnetic simulations, we consider a monolayer or bilayer model with open boundary conditions in the *x* and *y* dimensions. For the monolayer model, the length, width, and thickness of the model are set to 50, 50, and 4 uc, respectively, where 1 uc (i.e., unit cell) is equal to 0.4 nm. For the bilayer model, the length, width, and thickness of each ferromagnetic layer (SRO) are set to 50, 50, and 4 uc, respectively. The spacing between the two ferromagnetic layers, that is, the thickness of the BTO layer, is set to 1, 4, 5, or 10 uc. The simulation parameters were selected to remain within the physically reasonable range for ultrathin SRO‐based heterostructures and interfacial chiral‐spin systems. The intrinsic magnetic parameters for the ferromagnetic layer (SRO) are: the exchange constant *A* = 5 pJ m^−1^, the DMI constant *D* = 1.0–3.0 mJ m^−2^, the saturation magnetization *M*_S_ = 0.19 MA m^−1^, and the perpendicular magnetic anisotropy (PMA) constant *K* = 0.3–0.7 MJ m^−3^ (the default being 0.5 MJ m^−3^) were chosen as a physically reasonable parameter window for ultrathin SRO‐based oxide heterostructures, guided by previous reports on BTO/SRO, SIO/SRO, and micromagnetic DMI‐stabilized skyrmion systems (Table S1) [7, 10, 22, 54, 55, 56, 57, 58]. For the spacer layer (BTO), the saturation magnetization is set to zero. In the bilayer model, the top and bottom ferromagnetic layers (SRO) are coupled via stray field, that is, the long‐range dipolar interactions between the top and bottom layers.

The magnetization dynamics in micromagnetic simulations is controlled by the Landau‐Lifshitz‐Gilbert (LLG) equation, given as

(15) $$\partial_{t}m=-\gamma_{0}m\times h_{\mathrm{eff}}+\alpha \left(m\times \partial_{t}m\right)$$

where ***m*** is the reduced magnetization, *t* is the time, γ_0_ =  2.211 × 10^5^ m A^−1^ S^−1^ is the absolute gyromagnetic ratio, and α = 0.3 is the Gilbert damping parameter. The effective field $h_{\mathrm{eff}}=-\frac{1}{\mu_{0}M_{S}}\cdot \frac{\delta \varepsilon }{\delta m}$, where ε denotes micromagnetic energy density. In this work, we included the ferromagnetic exchange interaction, DMI, PMA, demagnetization, and applied magnetic field terms, and the total energy density is expressed as

(16) $$\begin{array}{ccc} \varepsilon & = & A{\left(\nabla m\right)}^{2}+D\left[m_{z}\left(m\cdot \nabla \right)-\left(\nabla \cdot m\right)m_{z}\right]\\ & & -\;K{\left(n\cdot m\right)}^{2}-\frac{M_{S}}{2}\left(m\cdot B_{d}\right)-M_{S}\left(m\cdot B_{e}\right) \end{array}$$

where *A*, *D*, and *K* stand for the ferromagnetic exchange, DMI, and PMA constants, respectively. ***B***_d_ is the demagnetization field, and ***B***_e_ is the applied magnetic field. For each relaxation simulation at zero external field, the initial spin configuration considered in the model is a ferromagnetic state, a random spin configuration, or a magnetic skyrmion‐like state.

## Author Contributions

**Sagar E. Shirsath**: Writing – original draft, investigation, formal analysis. **Yunlong Sun**: investigation, formal analysis, writing – review and editing. **Xichao Zhang**: writing – review and editing, formal analysis. **Ji Zhang**: investigation, writing – review and editing, formal analysis. **Akash V. Fulari**: writing – review and editing, investigation. **Zizheng Song**: writing – review and editing, formal analysis, investigation. **Yee Yan Tay**: writing – review and editing, formal analysis. **Yin Yao**: investigation, formal analysis. **Zibin Chen**: writing – review and editing, investigation, formal analysis. **Sean Li**: writing – review and editing, formal analysis. **Xiaoxi Liu**: writing – review and editing, formal analysis, investigation, supervision. **Danyang Wang**: methodology, investigation, writing – review and editing, funding acquisition, formal analysis, project administration, supervision, resources.

## Conflicts of Interest

The authors declare no conflicts of interest.

## Supporting information




**Supporting File**: advs78126‐sup‐0001‐SuppMat.docx.

## Data Availability

The data that support the findings of this study are available from the corresponding author upon reasonable request.
