# Supplementary material for: Interfacial Asymmetry and Surface Roughness Enhanced Topological‐Hall‐Like Transport in SRO/BTO/SRO Trilayers
Source: Adv Sci (Weinh). 2026 Oct 6:e78126. Online ahead of print. doi: 10.1002/advs.78126 (PMC13643100; doi:10.1002/advs.78126)
Supplement: Supplementary file 1 [file ADVS-9999-e78126-s001.docx]

**Supporting Information**

**Interfacial Asymmetry and Surface Roughness Enhanced Topological-Hall-Like Transport in SRO/BTO/SRO Trilayers**

Sagar E. Shirsath^1^, Yunlong Sun^1^, Xichao Zhang^2^, Ji Zhang^1^, Akash V. Fulari^3^, Zizheng Song^4,5^, Yee Yan Tay^6^, Yin Yao^7^, Zibin Chen^4,5^, Sean Li^1^ , Xiaoxi Liu^8,^*, Danyang Wang^1,^*

*^1^School of Materials Science and Engineering, The University of New South Wales, Sydney, NSW 2052, Australia*

*^2^Department of Electronic and Computer Engineering, The Hong Kong University of Science and Technology, Hong Kong, China*

*^3^ Symbiosis Centre for Nanoscience and Nanotechnology, Symbiosis International (Deemed University), Pune, MS, India*

*^4^ State Key Laboratory of Ultra-Precision Machining Technology, Department of Industrial and Systems Engineering, The Hong Kong Polytechnic University, Kowloon, Hong Kong, China*

*^5^ Research Institute for Advanced Manufacturing, Department of Industrial and Systems Engineering, The Hong Kong Polytechnic University, Hong Kong, China*

*^6^ Facility for Analysis, Characterisations, Testing and Simulations (FACTS), Nanyang Technological University, 61 Nanyang Drive, Singapore 637335, Singapore*

*^7^Electron Microscope Unit, Mark Wainwright Analytical Centre UNSW Sydney, The University of New South Wales, Sydney, NSW 2052, Australia*

*^8^Department of Electrical and Computer Engineering, Shinshu University, Nagano 380-8553, Japan*

**Bilayer** BaTiO_3_ (BTO) 10 u.c./SrRuO_3_ (SRO) 4 u.c./ SrTiO_3_ (STO) substrate

BTO

SRO

**Trilayer** SrRuO_3_ (SRO) 4 u.c./ BaTiO_3_ (BTO) 10 u.c./SrRuO_3_ (SRO) 4u.c./ SrTiO_3_ (STO) substrate

SRO

BTO

SRO

**Figure S1: Schematic illustration of the heterostructure architectures.** Cross-sectional diagrams detailing the layer sequence and unit cell (u.c.) thicknesses for the BaTiO_3_(10 u.c.)/SrRuO_3_ (4 u.c.) bilayer and the SrRuO_3_ (4 u.c.)/BaTiO_3_ (10 u.c.)/SrRuO_3_(4 u.c.) trilayer grown on SrTiO_3_ (STO) substrates .

**Figure S2: Atomic-scale structural imaging of the trilayer.** Cross-sectional high-angle annular dark-field (HAADF) scanning transmission electron microscopy (STEM) images of the SRO/BTO/SRO trilayer. The right panel displays the corresponding atomic column intensity profile, clearly resolving the alternating Ba/Ti and Sr/Ru sequences across the interfaces to confirm the structural integrity of the stack.

**Figure S3: Elemental composition across the trilayer interfaces.** Energy-dispersive X-ray spectroscopy (EDX) elemental mapping of the SRO/BTO/SRO heterostructure . The spatial distribution maps and line profiles for Oxygen (O), Titanium (Ti), Strontium (Sr), Ruthenium (Ru), and Barium (Ba) confirm minimal interdiffusion and sharp chemical boundaries between the ferromagnetic and ferroelectric layers

**Figure S4: In-situ growth monitoring of SRO/BTO/SRO trilayer via RHEED.** Reflection high-energy electron diffraction (RHEED) patterns and corresponding intensity oscillations recorded during the laser molecular beam epitaxy (LMBE) growth. The panels display the RHEED patterns for the bare STO substrate, the bottom STO/SRO interface, the STO/SRO/BTO intermediate surface, and the final STO/SRO/BTO/SRO top surface, respectively. The distinct intensity oscillations confirm a high-quality, layer-by-layer epitaxial growth mode.

**Figure S5: In-situ growth monitoring of SRO/BTO bilayer via RHEED.** Reflection high-energy electron diffraction (RHEED) patterns and corresponding intensity oscillations recorded during the laser molecular beam epitaxy (LMBE) growth. The panels display the RHEED patterns for the bare STO substrate, the bottom STO/SRO interface, and the STO/SRO/BTO. The distinct intensity oscillations confirm a high-quality, layer-by-layer epitaxial growth mode.

**Figure S6: Sensitivity of micromagnetic relaxation to the initial magnetic configuration.** Simulated steady-state magnetic configurations in a 4 u.c. thick SRO layer (50 × 50 u.c. lateral dimensions) as a function of the Dzyaloshinskii-Moriya interaction (DMI) strength. To verify the true energetic minimum, the system was allowed to relax from three distinct starting conditions: a uniformly magnetized ferromagnetic state (FM), a random noise state (RA), and a predefined skyrmion state (SK). For the SRO layer, the simulations were carried out with exchange stiffness *A* = 5pJ m^−1^, saturation magnetization *M*_S_ = 0.19 MA m^−1^, DMI strength D = 1.0-3.0 mJ m^−2^, and anisotropy K = 0.5 MJ m^−3^, while multiple initial magnetic states, including ferromagnetic, random, and skyrmion states, were tested to examine the stability of the relaxed solutions.

**Figure S7: Relative energies of the tested relaxed magnetic configurations.** Calculated total magnetic energy as a function of DMI strength (D) for the relaxed configurations corresponding to the FM, RA, and SK initial states shown in Figure S6. The crossover points indicate the critical D values above which the non-collinear skyrmion state becomes the global energy minimum.

50 uc

50 uc

4 uc

SRO

**Figure S8: Anisotropy and DMI parameter sweep.** Micromagnetic phase diagrams illustrating the stabilized spin textures in the SRO monolayer architecture as a function of varying uniaxial anisotropy (K = 0.30 - 0.70 MJ m^−3^) and DMI strength (D = 1.0 - 3.0 mJ m^2^). The calculations show persistence of noncollinear relaxed configurations over a finite region of the tested K−D parameter space. These phase maps demonstrate the model’s parameter sensitivity and physical plausibility but do not identify the experimentally realized magnetic configuration or exclude a multichannel anomalous Hall contribution.

**Figure S9: Anisotropy and DMI parameter sweep.** Micromagnetic phase diagrams illustrating the stabilized spin textures in the bottom and top SRO layers of the trilayer architecture as a function of varying uniaxial anisotropy (K = 0.30 - 0.70 MJ m^−3^) and DMI strength (D = 1.0 - 3.0 mJ m^2^). The calculations show persistence of noncollinear relaxed configurations over a finite region of the tested K−D parameter space. These phase maps demonstrate the model’s parameter sensitivity and physical plausibility but do not identify the experimentally realized magnetic configuration or exclude a multichannel anomalous Hall contribution.

**Supplementary Note S1: Scope and limitations of the micromagnetic model**

The simulations describe magnetic-energy relaxation and do not calculate electrical transport or anomalous Hall conductivity. The upper and lower SRO layers are not assigned experimentally determined layer-specific anomalous Hall coefficients or carrier densities. Structural disorder and surface roughness are represented only indirectly through the tested parameter ranges. Consequently, the simulations test whether the trilayer architecture can support noncoplanar configurations, but they cannot distinguish such configurations from a multichannel AHE origin of the measured Hall anomaly.

**Micromagnetic parameters**

The micromagnetic parameters used in the present calculations were selected as literature-guided representative values and physically reasonable sensitivity ranges for ultrathin SrRuO_3_-based oxide heterostructures, rather than as quantities uniquely fitted to the measured Hall curves. Previous SrRuO_3_ micromagnetic studies have employed exchange-stiffness values of approximately 1.8–2.0 pJ m^−1^, saturation magnetization near 0.20 MA m^−1^, and magnetocrystalline-anisotropy energy densities of approximately 0.64–1.2 MJ m^−3^.[1-4] These reports establish the relevant order of magnitude for the magnetic parameters, while also showing that their effective values depend strongly on strain, thickness, interface termination, crystallographic orientation, and temperature.

Accordingly, the present value A = 5 pJ m^−1^ was adopted as a fixed model value within the same few-pJ m^-1^ order of magnitude; sensitivity to was not independently examined in the present calculations. The selected Ms = 0.19 MA m^−1^ and the anisotropy range K = 0.3–0.7 MJ m^−3^ are consistent with reported SrRuO_3_ magnetic scales.[1-4] The interfacial DMI was varied from D = 1.0 to 3.0 mJ m^−2^ as an exploratory parameter window relevant to ultrathin SrRuO_3_ heterostructures in which broken inversion symmetry and strong spin–orbit coupling generate interface-driven chiral magnetic interactions.[1, 5-7] Thus, the D and K sweeps test the accessibility and robustness of noncollinear relaxed configurations; they are not presented as sample-specific measurements or as a unique fit to the experimental Hall response.

**Table S1.** Micromagnetic parameters used in the simulations and their physical/literature basis. The parameters are literature-guided model inputs and sensitivity ranges, not uniquely fitted experimental quantities.

| **Parameter** | **Value used** | **Region** | **Physical and literature basis** | **Role in the model** |
| --- | --- | --- | --- | --- |
| **Exchange stiffness, A** | 5 pJ m^-1^ | SRO | Representative value in the few-pJ m^-1^ regime. Related SRO micromagnetic studies used A ≈ 1.8–2.0 pJ m^-1^.[1, 2] The present value is a rounded modelling choice rather than a direct measurement. | Penalizes rapid spatial variation of the magnetization and sets the characteristic magnetic-length scale. |
| **Saturation magnetization, Mₛ** | 0.19 MA m^-1^ | SRO | Consistent with the ≈0.20 MA m^-1^ SRO value used in a first-principles-informed micromagnetic model and with the established magnetic scale of SRO thin films.[1, 4] | Determines the Zeeman and magnetostatic/demagnetization energies. |
| **Interfacial DMI, D** | 1.0–3.0 mJ m^-2^ | SRO interfaces | Exploratory sensitivity range for an interface-induced chiral interaction. Broken inversion symmetry and strong spin–orbit coupling are established routes to DMI and noncoplanar textures in BTO/SRO and SIO/SRO heterostructures.[5-8] | Favors chiral, noncollinear magnetization configurations. |
| **Perpendicular anisotropy, K** | 0.3–0.7 MJ m^-3^; default 0.5 MJ m^-3^ | SRO | Sensitivity window centered on the reported SRO anisotropy scale. Values near 0.64 MJ m^-3^ have been used/reported for SRO, while larger values can occur depending on orientation, strain, and temperature.[1-4] | Controls the preferred magnetization direction and competes with DMI and demagnetization. |
| **Saturation magnetization** | 0 | BTO spacer | BTO is treated as a nonmagnetic ferroelectric spacer. Its role is incorporated through geometry, interfacial symmetry breaking, and indirect boundary effects rather than a direct magnetic moment.[6] | Prevents a direct ferromagnetic contribution from the BTO spacer. |
| **BTO spacer thickness** | 1, 4, 5, and 10 u.c. | BTO spacer | Computational thickness sweep; 10 u.c. corresponds to the experimentally investigated trilayer, whereas the thinner spacers are comparison models. | Tests the influence of magnetic-layer separation and dipolar/indirect interlayer effects. |
| **Gilbert damping, α** | 0.3 | SRO | Numerical relaxation parameter selected to accelerate convergence to a stationary state; it is not used as a fitted dynamic material constant. | Controls the relaxation rate in the Landau–Lifshitz–Gilbert equation. |

The parameter selection in Table S1 was designed to test whether the experimentally realized trilayer architecture can support noncollinear magnetic configurations over a finite model-parameter range. The persistence of such configurations across different initial states and across the D–K parameter sweep demonstrates that the calculated result is not restricted to one isolated parameter point. Nevertheless, the simulations do not constitute a quantitative reconstruction of the exact experimental spin texture. They do not calculate layer-resolved anomalous Hall conductivities or explicitly model two parallel electrical-transport channels; consequently, they cannot exclude a multichannel anomalous Hall contribution to the measured hump-like Hall response. The simulations should therefore be interpreted as a physical-consistency and robustness test, rather than as unique microscopic proof of a skyrmion or other specific noncoplanar magnetic state.

References

[1] A. Schäffer, L. Chotorlishvili, I. Maznichenko, A. Ernst, K. Dörr, I. Mertig, J. Berakdar, Element specific hysteresis of La0. 7Sr0. 3MnO3—SrRuO3 (LSMO-SRO) heterostructures, APL Materials, 6 (2018).

[2] Q. Qin, S. He, W. Lin, H.Y. Yoong, L. Liu, M. Li, S.J. Pennycook, W. Xiao, P. Yang, W. Song, Interfacial antiferromagnetic coupling between SrRuO3 and La0. 7Sr0. 3MnO3 with orthogonal easy axis, Phys. Rev. Mater, 2 (2018) 104405.

[3] A. Kanbayasi, Magnetocrystalline anisotropy of SrRuO3, J. Phys. Soc. Jpn., 41 (1976) 1879-1883.

[4] G. Koster, L. Klein, W. Siemons, G. Rijnders, J.S. Dodge, C.-B. Eom, D.H. Blank, M.R. Beasley, Structure, physical properties, and applications of SrRuO 3 thin films, Rev. Mod. Phys., 84 (2012) 253-298.

[5] J. Matsuno, N. Ogawa, K. Yasuda, F. Kagawa, W. Koshibae, N. Nagaosa, Y. Tokura, M. Kawasaki, Interface-driven topological Hall effect in SrRuO3-SrIrO3 bilayer, Science advances, 2 (2016) e1600304.

[6] L. Wang, Q. Feng, Y. Kim, R. Kim, K.H. Lee, S.D. Pollard, Y.J. Shin, H. Zhou, W. Peng, D. Lee, Ferroelectrically tunable magnetic skyrmions in ultrathin oxide heterostructures, Nature materials, 17 (2018) 1087-1094.

[7] K.-Y. Meng, A.S. Ahmed, M. Bacani, A.-O. Mandru, X. Zhao, N. Bagués, B.D. Esser, J. Flores, D.W. McComb, H.J. Hug, Observation of nanoscale skyrmions in SrIrO3/SrRuO3 bilayers, Nano Lett., 19 (2019) 3169-3175.

[8] M. Cuoco, A. Di Bernardo, Materials challenges for SrRuO3: From conventional to quantum electronics, APL Materials, 10 (2022).
